# Generating Detailed Spectral Libraries for Canine Proteomes Obtained from Serum and Urine

**DOI:** 10.1038/s41597-023-02139-6

**Published:** 2023-04-27

**Authors:** Hee-Sung Ahn, Jeonghun Yeom, Jiyoung Yu, Yumi Oh, JeongYeon Hong, Minjung Kim, Kyunggon Kim

**Affiliations:** 1 Convergence Medicine Research Center, Asan Institute for Life Sciences, Asan Medical Center, Seoul, 05505 Republic of Korea; 2 Clinical Proteomics Core Laboratory, Convergence Medicine Research Center, Asan Medical Center, Seoul, 05505 Republic of Korea; 3 Prometabio Research Institute, Prometabio co., ltd., Gyeonggi-do, 12939 Republic of Korea; 4 Asan Institute for Life Sciences, Asan Medical Center, Seoul, 05505 Republic of Korea; 5 Department of Biomedical Sciences, University of Ulsan College of Medicine, Seoul, 05505 Republic of Korea; 6 Department of Research and Development, Mjbiogen, Seoul, 04788 Republic of Korea; 7 Bio-Medical Institute of Technology, Asan Medical Center, Seoul, 05505 Republic of Korea

**Keywords:** Proteomics, Biomarkers

## Abstract

Domestic dogs (*Canis lupus familiaris*) are popular companion animals. Increase in medical expenses associated with them and demand for extending their lifespan in a healthy manner has created the need to develop new diagnostic technology. Companion dogs also serve as important animal models for non-clinical research as they can provide various biological phenotypes. Proteomics have been increasingly used on dogs and humans to identify novel biomarkers of various diseases. Despite the growing applications of proteomics in liquid biopsy in veterinary medicine, no publicly available spectral assay libraries have been created for the proteome of canine serum and urine. In this study, we generated spectral assay libraries for the two-representative liquid-biopsy samples using mid-pH fractionation that allows in-depth understanding of proteome coverage. The resultant canine serum and urine spectral assay libraries include 1,132 and 4,749 protein groups and 5,483 and 25,228 peptides, respectively. We built these complimentary accessible resources for proteomic biomarker discovery studies through ProteomeXchange with the identifier PXD034770.

## Background & Summary

Disease diagnosis is important for the welfare of companion dogs, including enabling proper treatment and reducing medical expenses that may arise in the future^1–3^. Biomarker-driven research and development improves both the sensitivity and specificity of diagnosis in various dog diseases, which can contribute towards extending the lifespan of dogs^4–7^. In addition, short-lived dogs living in the human-like circumstances form an animal model suitable for understanding human diseases and support translational discoveries^8^. Longitudinal studies that use multiomics for analyzing different types of samples collected throughout the life cycle from various breeds of dogs are recently being conducted^8–10^. Collection of such large veterinary medical data sets provide an opportunity to find more information about health and diseases in dogs^11^.

The proteomics approach derived from mass spectrometry (MS) has been useful in the discovery of novel protein biomarkers by aiding in acquisition of quantitative protein information from complex biological samples;^12–16^ it enables biomarker-based disease diagnosis, prognosis, and therapeutic monitoring. Deep proteomic spectral libraries created using fractionated samples are built through data-dependent acquisition (DDA) mass spectrometry and are mainly employed to analyze data-independent acquisition (DIA)-mass spectrometry of unfractionated samples^17^. Comprehensive spectral libraries are required for robust quantification of maximum number of proteins in complex samples.

Shotgun proteomics has been used in searching for protein biomarkers using canine serum and urine samples. Several studies on dilated cardiomyopathy^18^, dyssynchrony heart failure^19^, mitral valve disease^20^, chronic valve disease^21^, osteoarthritis^22^, babesiosis^23–25^, ehrlichiosis^26^, leishmaniasis^27–29^, hemangiosarcoma^30^, high-grade multicentric lymphoma^31^, osteosarcoma^32^, and mammary tumors have utilized serum samples^33^, whereas studies on X-linked hereditary nephropathy^34^, chronic kidney disease^35,36^, dirofilariasis^37^, transitional cell carcinoma^38^, and venomous bite from a European viper have utilized urine samples^39^.

Publicly available spectral libraries for canine serum and urine proteome are necessary for conducting studies to discover the uses of new disease biomarkers. Serum contains certain high-concentration proteins (i.e., albumin) that make it difficult to measure low-concentration proteins using liquid chromatography-MS (LC-MS), which remains the primary reason that more than 1,000 canine proteins have not yet been identified in urine^40^. Therefore, individual spectral libraries for serum and urine are needed and could help in identifying more proteins.

Comprehensive serum and urine spectral libraries were built from 24 fractionated serum and urine proteome using a high-resolution Orbitrap mass spectrometer. The serum protein library includes 5,483 peptides mapped to 1,132 serum proteins (Supplementary Table 1). The urine protein library has 25,228 peptides mapped to 4,749 urinary proteins (Supplementary Table 2). We analyzed the characteristics of the two libraries using DIALib-QC^41^. Spectral libraries can be used to interpret DIA data collected on other instruments and for qualitative and quantitative analysis of peptides and proteins through spectral matching with DDA data. We deposited raw MS data from the instrument and spectral libraries with ProteomeXchange^42^ Consortium (http://proteomecentral.proteomexchange.org) through the Proteomics Identification Database (PRIDE)^43^ and allied repository with identifier PXD034770.

## Methods

### Materials and reagents

The materials and reagents used in this study were purchased from various sources: HPLC grade acetonitrile and water from Avantor (Radnor, PA, USA); LC-MS grade formic acid (FA) and bicinchoninic acid (BCA) assay kit from Thermo Fisher Scientific (Pittsburgh, PA, USA); MS-grade trypsin/lys-C Mix from Promega (Madison, WI, USA); suspension-trapping (S-trap) digestion kit from Protifi (Huntington, NY, USA); and triethyammonium bicarbonate (TEAB) from Sigma-Aldrich (Burlington, MA, USA).

### Canine serum and urine collection

The features of the dogs sampled in this study are summarized in Table 1. Data was collected from 82 dogs belonging to 19 breeds consisting of 44 neutered males, 28 spayed females, 5 females, and 5 males. Serum and urine samples were collected from 12 dogs with acute pancreatitis, 12 dogs with chronic pancreatitis, 18 dogs with lymphoma, and 40 healthy dogs. Blood was drawn from the cephalic vein, and sera were separated by centrifuging for 10 min. Urine samples were collected by free catch. The sample procedures followed the guidelines of the Institutional Animal Care and Use Committee (IACUC) of Seoul National University (Approval number, SNU-200701-6-2; Approval date, 2020.09.28)^44^.

**Table 1:** Study population of 19 breeds of 82 dogs. Average age, sex, and disease status were represented.

Breed	N	Average age (years)	Sex				Disease status			
			MN	FS	F	M	Healthy	Acute pancreatitis	Chronic pancreatitis	Lymphoma
Bichon Frise	1	2.0	—	—	1	—	1	—	—	—
Chihuahua	8	6.6	1	6	—	1	5	1	2	—
Cocker Spaniel	3	9.7	3	—	—	—	—	—	—	3
Coton de Tulear	1	11.0	1	—	—	—	—	—	1	—
Dachshunds	3	5.3	2	—	1	—	1	—	—	2
English Cocker Spaniel	1	3.0	1	—	—	—	1	—	—	—
French Bulldog	1	0.5	1	—	—	—	1	—	—	—
Golden Retriever	1	0.6	1	—	—	—	1	—	—	—
Labrador Retriever	2	1.2	—	—	—	2	1	—	—	1
Maltese	21	7.2	12	7	1	1	14	3	1	3
Maltipoo	2	1.7	2	—	—	—	2	—	—	—
Pomeranian	4	6.8	1	2	1	—	1	2	—	1
Poodle	10	7.0	5	5	—	—	1	5	2	2
Poongsan dog	1	0.9	—	1	—	—	1	—	—	—
Shetland Sheepdog	1	3.0	—	1	—	—	1	—	—	—
Shih Tzu	8	8.3	5	3	—	—	4	—	1	3
Toy Poodle	1	1.0		1	—	—	1	—	—	—
Welsh Corgi	2	2.3	1	—	—	1	2	—	—	—
Yorkshire Terrier	11	9.6	8	2	1	—	2	1	5	3

M: male, MN: male neutered, F: female, FS: female spayed.

### Serum protein sample preparation

Serum samples (40 μL) were passed through a MARS14 column (100 × 4.6 mm; Agilent Technology, Palo Alto, CA, USA) on a binary HPLC system (20 A Prominence; Shimadzu, Tokyo, Japan) to reduce 14 high serum proteins; the unbound part was quantified by BCA assay and only 100 μg of it was freeze-dried with a cold trap (CentriVap Cold Traps; Labconco, Kansas City, MO, USA).

### Urine sample preparation

One milliliter of urine was lyophilized using a cold trap. Dried urine samples were resuspended in 100 μL of 5% SDS in 50 mM TEAB (pH 8.5) and 100 μL of protein was lyophilized after BCA quantification.

### Peptide sample preparation

One hundred microgram of dried serum or urine sample was reconstituted with 400 μL of 5% SDS in 50 mM TEAB (pH 8.5). Dithiothreitol was added to a final concentration of 20 mM denatured sample, and the reaction was carried out at 95 °C for 10 min. The reduced sample was then placed in iodoacetamide at a final concentration of 40 mM and incubated for 30 min at 25 °C in the dark. Using 1.2% phosphoric acid, samples were attached to S-Trap mini columns (ProtiFi, Farmingdale, NY, USA, Cat. No: CO2-mini-80). Following the manufacturer’s protocol, we performed S-trap proteolysis by adding 10 μg Lys-C/trypsin mixture and incubating at 37 °C for 16 h^45^. The digested peptide mixture was freeze-dried with a cold trap and stored at −80 °C.

### Mid-pH reverse-phase fractionation

Pooled digested serum and urine peptide mixtures were prepared from the 25 μg of 82 individual canine digested serum and urine peptides, the concentrations of which were measured with a UV/Vis spectrophotometer (NanoDrop One, Thermo Fisher Scientific, Waltham, MA, USA) at a wavelength of 280 nm, with the sample type option set to “1 Abs = 1 mg/mL”. Their separation was performed on an XBridge C-18 column (4.6 mm i.d. × 250 mm length; pore size, 130 Å; and particle size, 5 μm; Waters Corporation, USA), using a Shimadzu Prominence HPLC instrument (Shimadzu) at a flow rate of 0.5 mL/min for 120 min. We used 10 mM TEAB (pH 8.5) in HPLC water as mobile phase A and 10 mM TEAB (pH 8.5) in 90% acetonitrile and 10% HPLC water as mobile phase B. The sample was dissolved in 80 μL of mobile phase A and then injected into a 100 μL sample loop. A gradient of 5-5% B for 8 min, 5–40% B for 57 min, 40–44% B for 5 min, 44–60% B for 4 min, 60-60% B for 14 min, 60-5% B for 2 min and 5-5% B for 30 min were applied. From 8 to 94.4 min, the eluents were collected in increments of 0.45 mL in a 96-well plate on an FRC-10A of the Shimadzu Prominence (Shimadzu). The resulting 96 fractions were mixed in a concatenate manner to form 24 fractions (Table 2). Each fraction was dried and stored at –80 °C.

**Table 2:** Sample overview.

Sample type	Fractions	Library name
Serum	24	DogSerum_Library
Urine	24	DogUrine_Library

Sample types, numbers of fractions after mid-pH reverse phase fractionation, and names of the generated spectral assay libraries were included.

### Nano LC-MS/MS

Peptide mixtures were separated by using the Dionex UltiMate 3000 RSLC nano system (Thermo Fisher Scientific, Waltham, MA, USA). The dried sample was resuspended in 0.1% formic acid to a concentration of 1 μg/μL, and 5 μL of which was loaded on a C18 Pepmap trap column (20 mm × 100 μm i.d., 5 μm, 100 Å; Thermo Fisher Scientific) and separated with an Acclaim™ Pepmap 100 C18 column (500 mm × 75 μm i.d., 3 μm, 100 Å; Thermo Fisher Scientific) over 200 min (250 nL/min) using a 0–48% acetonitrile gradient in 0.1% formic acid and 5% DMSO for 150 min at 50 °C. The LC was connected with a Q Exactive HF-X mass spectrometer (Thermo Fisher Scientific) with an EASY-Spray nano-ESI source. In a data-dependent mode, mass spectra were obtained with an automatic switch between a full scan with top 20 data-dependent MS/MS scans. Resolution was set to 60,000 at m/z 200, and target value of 3,000,000 for MS scan type was selected. The ion target value for MS/MS was set at 100,000 with a resolution of 15,000 at m/z 200. The maximum ion injection time was set to 100 ms for the full scan and 50 ms for MS2 scan. Isolation width was 1.7 m/z, and normalized collision energy was set at 27. Dynamic exclusion for measurements of repeated peptides was set for 40 s.

### Data processing

Software Spectronaut v14 (Biosynosis, Switzerland) was used for data processing with default settings to build spectral libraries with the UniProt SwissProt and TrEMBL integrated database (*Canis lupus familiaris* (Taxon ID 9615); downloaded on 05/04/2022; 113,977 entries including both reviewed (838) and unreviewed (113,139) entries). N-terminal acetylation and methionine oxidation were set as variable modifications, and cysteine carbamidomethylation was set as a fixed modification. We set the false discovery rate (FDR) <1% at the peptide-spectrum match (PSM), peptide, and protein levels, respectively. Spectral libraries for canine serum and urine proteomes were created using 24-DDA raw mass spectrometry data.

## Data Records

The two spectral libraries (.xls and .kit) produced from raw mass spectrometry data (.raw) and DIALib-QC evaluation reports for both were saved to the ProteomeXchange Consortium through PRIDE with the dataset identifier PXD034770^46^. The mass spectrometry files were named “Dog(name of the sample type)-(fraction number).raw”. The spectral libraries were named “Dog(name of the sample type)_Library.kit” and imported to Spectronaut software. Individual DIALib-QC evaluation reports for the two spectral libraries were included in this process.

## Technical Validation

High-level standard assay libraries are needed for accurate quantification of peptides and proteins. To get as many peptides as possible, a library was constructed with DDA-MS datasets obtained by reducing sample complexity through mid-pH separation. The search engine performed FDR calculation through target-decoy method using Biognosys’s Pulsar, and the levels of PSM, peptide, and protein group were controlled to 1% or less. To build the spectral library for the serum and urine proteome, library-wide FDR controls were applied to <1% at the three levels.

**Fig. 1:**
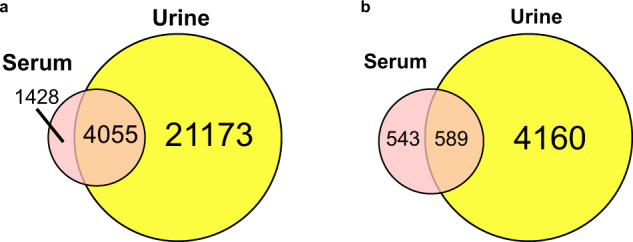
Number of constituent peptides and proteins in the canine serum and urine proteome spectral libraries. (**a**) Venn diagram of the number of peptides in two spectral libraries. (**b**) Venn diagram of the number of proteins in two spectral libraries.

The canine serum spectral library contained 97,888 transitions, identifying 6,159 peptide precursors and representing 5,483 stripped peptides and 1,132 protein groups. The canine urinary spectral library included 347,749 transitions identifying 27,922 peptide precursors interpreting 25,228 stripped peptides and 4,749 protein entries. Comparison between the spectral library for each dog specimen showed that 589 protein groups were common. In addition, there were 543 and 4,160 protein groups that were uniquely identified in canine serum and urine, respectively (Fig. 1a). A total of 4,055 peptides were found to be common between the spectral library for each dog specimen. In addition, there were 1,428 and 21,173 peptides that were uniquely identified in canine serum and urine, respectively (Fig. 1b).

**Fig. 2:**
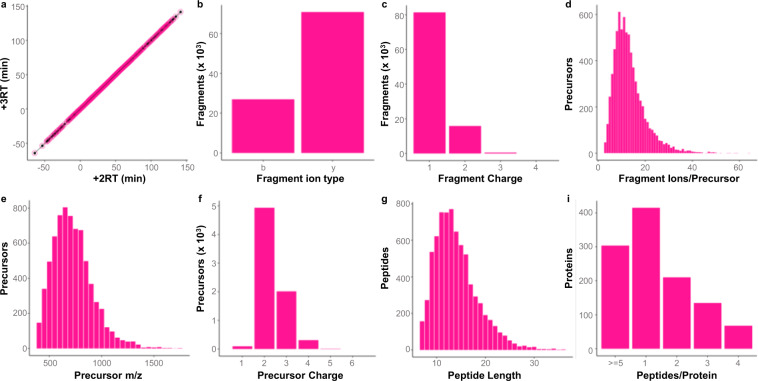
Characteristics of the canine serum proteome spectral assay library. (**a**) Scatter plot of the retention times (RTs) of the 2+ and 3+ charge ion states of the corresponding peptide. (**b**) Number of detected fragment ion type (b- and y-ion). (**c**) Histogram of the charge states of the detected fragment ions. (**d**) Frequency of precursors as a function of the number of fragment ions per precursor. (**e**) Histogram of precursors by precursor m/z values over the entire range of mass-to-charge ratio detected. (**f**) Histogram of the charge states of the detected precursor ions. (**g**) Frequency of peptides along sequence length. (**h**) Frequency of proteins as a function of number of peptides per protein.

We evaluated the quality and features of the canine serum proteome library by DIALib-QC. When contrasting the retention times (RTs) between the 2+ and 3+ ion charge states of precursors with the identical peptide, the two RTs with an R^2^ value of 1 showed almost identical values, proving the high level of chromatography (Fig. 2a). The normalized RT used here was calculated by the standard peptides in the spectral library. A higher proportion of y than of b ions (72.4 vs. 27.6%), that is previously known as higher-energy collisional dissociation fragmentation in Orbitrap^47^ (Fig. 2b). In addition, >99.4% of peptide fragment ions in the library have 1+ or 2+ charge states (Fig. 2c). Precursor ions containing five or fewer fragment ions do not account for 5.7% of the library (Fig. 2d). In the library, 98% of the precursors lie between 400 and 1,200 m/z (Figs. 2e), and 98.5% of the precursor states of charge range from +2 to +4 (Fig. 2f). More than 99.6% of all peptides in the library have 30 or fewer sequences (Fig. 2g). Proteins with two or more peptides possess 63.3% of the total amount, of which proteins with five or more peptides constitute 26.9% of the proteins in the library (Fig. 2h).

**Fig. 3:**
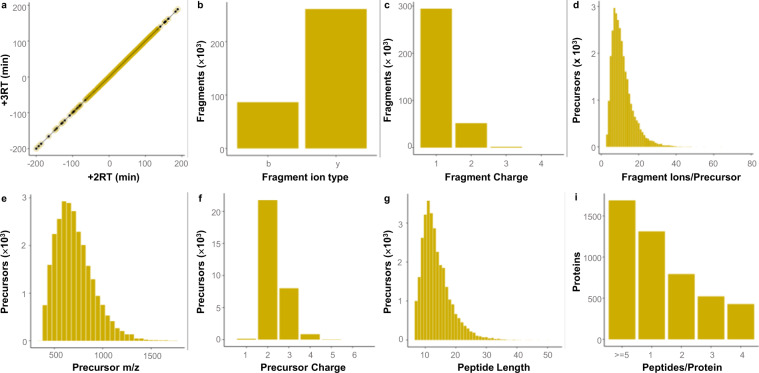
Characteristics of the canine urine proteome spectral assay library. (**a**) Scatter plot of the retention times (RTs) of the 2+ and 3+ charge ion states of the corresponding peptide. (**b**) Number of detected fragment ion type (b- and y-ion). (**c**) Histogram of the charge states of the detected fragment ions. (**d**) Frequency of precursors as a function of the number of fragment ions per precursor. (**e**) Histogram of precursors by precursor m/z values over the entire range of mass-to-charge ratio detected. (**f**) Histogram of the charge states of the detected precursor ions. (**g**) Frequency of peptides along sequence length. (**h**) Frequency of proteins as a function of number of peptides per protein.

We also applied DIALib-QC to appraise the canine urine proteome library. The RTs between the 2+ and 3+ ion charge states of the identical peptide show high positive correlation (R^2^ = 1, Fig. 3a). The proportion of b and y fragment ions are 75.1% and 24.9% (Fig. 3b). In the library, more than 99.5% of the fragment ions in charge states of 1+ or 2+ are included (Fig. 3c). As shown in Fig. 3d, peptides containing more than six fragment ions per the corresponding precursor accounted for beyond 89.6% of the total. In the library, 98.2% of the precursors belong to 400–1,200 m/z (Fig. 3e), and the charge state of 99.3% precursors range from +2 to +4 (Fig. 3f). More than 99.2% of all peptides in the library are in the sequence length range of 7–52 and have less than 30 mer in total (Fig. 3g). Proteins containing two or more peptides make up 72.3% of the total, of which proteins containing five or more peptides make up 35.5% of the proteins in the library (Fig. 3h).

## Supplementary information

Supplementary Table 1

Supplementary Table 2

## Data Availability

The commercial software Spectronaut generated a spectrum library, and free software DIALib-QC evaluated the constructed spectrum library, which is described in the Data Records section.
